# Hepatitis C virus screening of high-risk patients in a community hospital emergency department: Retrospective review of patient characteristics and future implications

**DOI:** 10.1371/journal.pone.0252976

**Published:** 2021-06-10

**Authors:** Ji Seok Park, Judy Wong, Hillary Cohen

**Affiliations:** 1 Department of Gastroenterology, Hepatology and Clinical Nutrition, Digestive Disease and Surgery Institute, Cleveland Clinic, Cleveland, Ohio, United States of America; 2 Department of Internal Medicine, Englewood Health, Englewood, New Jersey, United States of America; 3 Department of Emergency Medicine, Englewood Health, Englewood, New Jersey, United States of America; University of Cincinnati College of Medicine, UNITED STATES

## Abstract

**Background:**

Chronic hepatitis C virus infection (HCV) is a common infectious disease that affects more than 2.7 million people in the US. Because the emergency department (ED) can present an ideal opportunity to screen patients who may not otherwise get routine screening, we implemented a risk-based screening program for ED patients and established a system to facilitate linkage to care.

**Methods and findings:**

A risk-based screening algorithm for HCV was programmed to trigger an alert in Epic electronic medical record system. Patients identified between August 2018 and April 2020 in the ED were tested for HCV antibody reflex to HCV RNA. Patients with a positive screening test were contacted for the confirmatory test result and to establish medical care for HCV treatment. Patient characteristics including age, sex, self-awareness of HCV infection, history of previous HCV treatment, history of opioids use, history of tobacco use, and types of insurance were obtained.

A total of 4,525 patients underwent a screening test, of whom 131 patients (2.90%) were HCV antibody positive and 43 patients (0.95%) were HCV RNA positive, indicating that only 33% of patients with positive screening test had chronic HCV infection. The rate of chronic infection was higher in males as compared to females (1.34% vs 0.60%, p = 0.01). Patients with history of opioid use or history of tobacco use were found to have a lower rate of spontaneous clearance than patients without each history (opioids: 48.6% vs 72.0%, p = 0.02; tobacco: 56.6% vs 80.5%, p = 0.01). Among 43 patients who were diagnosed with chronic hepatitis C, 26 were linked to a clinical setting that can address chronic HCV infection, with linkage to care rate of 60.5%. The most common barrier to this was inability to contact patients after discharge from the ED.

**Conclusions:**

A streamlined EMR system for HCV screening and subsequent linkage to care from the ED can be successfully implemented. A retrospective review suggests that male sex is related to chronic HCV infection, and history of opioid use or history of tobacco use is related to lower HCV spontaneous clearance.

## Introduction

Chronic infection with hepatitis C virus (HCV) is a common infectious disease that affects more than 2.7 million people in the US [1]. Approximately 75–85% of newly infected hepatitis C patients develop chronic infection, a well-known risk factor for liver cirrhosis and eventual hepatocellular carcinoma [2]. To mitigate high mortality and morbidity associated with chronic hepatitis C, the Centers for Disease Control and Prevention (CDC) had recommended a risk-based screening test since 2012, which was targeted at persons born between 1945 and 1965, who had an HIV infection, who had past or present use of chronic hemodialysis, or who had resided in a high-prevalence country [3]. Against a backdrop of a persistently high public health burden, the CDC guideline was augmented in April 2020, recommending routine universal screening to all adults more than or equal to 18 years of age and all pregnant women except in settings where the prevalence of HCV infection is less than 0.1% [4]. Our intervention took place prior to the current guideline. Because the emergency department (ED) can present an ideal opportunity to screen patients who may not otherwise get routine screening, we implemented a risk-based screening program for ED patients and established a system to facilitate linkage to care (LTC).

## Materials and methods

### Study design and participants

**Fig 1:**
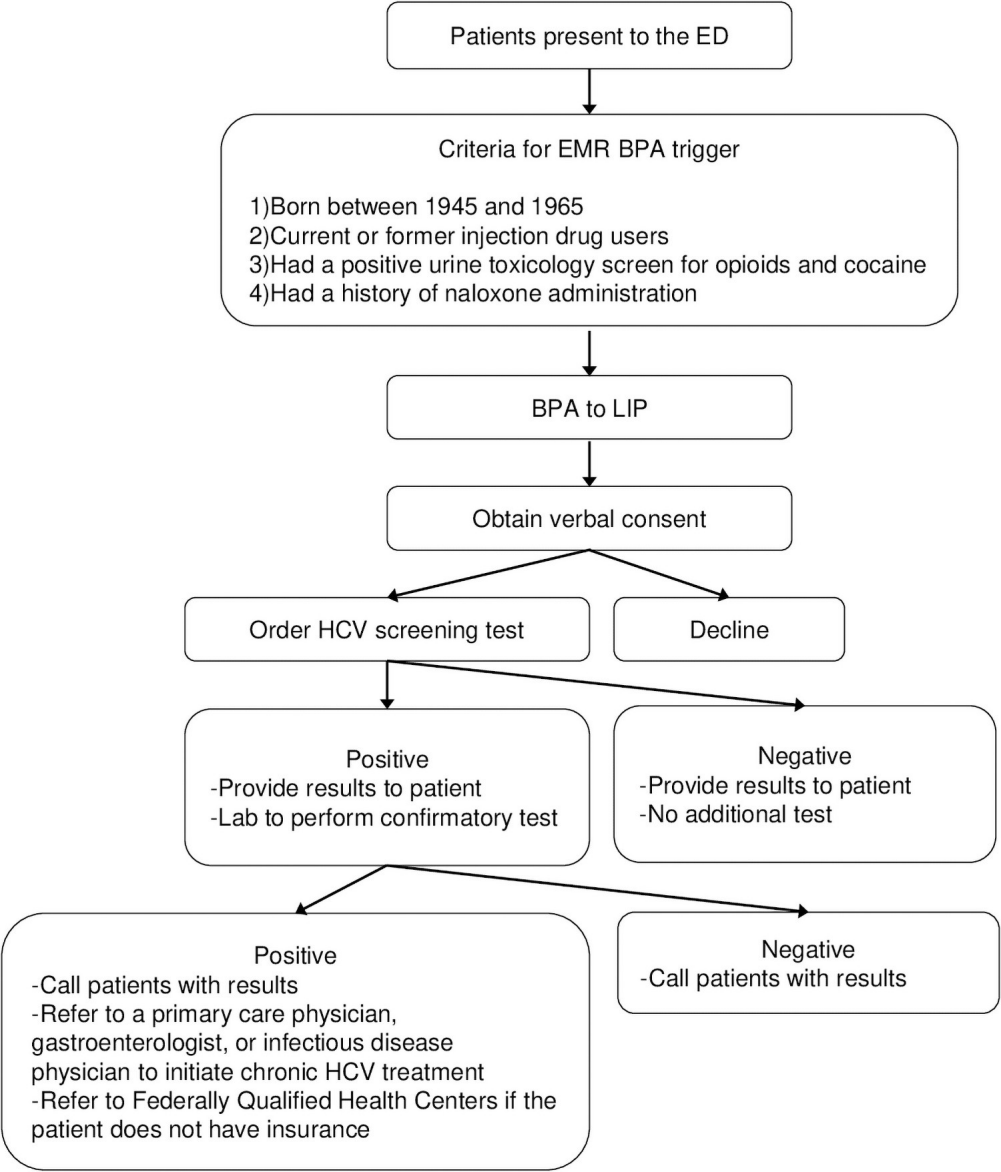
A risk-based HCV screening algorithm.

This was an observational study using patients’ data at a community hospital ED in Englewood, NJ, between August 1, 2018 and April 30, 2020. The annual ED census is more than 50,000 patients, and the department is located in an area that represents socioeconomically and racially diverse populations. Over a quarter of these patients are part of the “baby boomer” generation, making routine screening for chronic HCV infection, which has high prevalence in this population, particularly advantageous. In this setting, a risk-based HCV screening algorithm for HCV was programmed to trigger an alert in the Epic electronic medical record (EMR) system (Fig 1). Patients were tested for HCV antibody using a streamlined EMR system if they had consented verbally to testing. The consent was obtained by a licensed independent practitioner and documented in the EMR system. The criteria we used included patients who were born between 1945 and 1965, were current or former injection drug users, had a positive urine toxicology screen for opioids and cocaine, and had a history of naloxone administration before or after arrival at the ED. All positive screening tests were reflexively tested by the laboratory department for HCV RNA using the same sample. Social workers contacted all patients with a positive screening test as much as possible by automated certified letters and multiple phone calls for the confirmatory test result and to facilitate LTC. LTC was defined as successful when a patient was seen at the first appointment with a primary care physician or a hepatitis C specialist who could address chronic HCV infection, or when a patient was admitted to a residential substance use or mental health service that could address chronic HCV infection. The institutional review board at Englewood Health approved the study (E-18-730).

### Variables

Our primary variables of interest were HCV antibody test result, HCV RNA test result, and presence of successful LTC. Patient characteristics including age, sex, self-awareness of HCV infection, history of previous HCV treatment, history of any opioids use, history of tobacco use, and types of insurance were obtained. We made patient’s age a categorical variable by making three categories based on their birth year: 1965 and later, between 1945 and 1965, and before 1945. Other patient characteristics were dichotomous variables and types of insurance were categorized into private insurance, Medicare/Medicaid, and no insurance groups. All data were fully anonymized and recorded using Excel (Microsoft) by a research coordinator. The data were accessed by investigators on June 23, 2020.

### Analyses

Chi-square test was used to examine the relationships between categorical variables: between sex and chronic HCV infection; between sex and spontaneous viral clearance; between opioid use and chronic HCV infection; between tobacco use and chronic HCV infection; between age and opioid use; and between insurance status and LTC. Multiple logistic regression analysis was used for factors that were related to HCV spontaneous clearance. Data were analyzed with STATA (STATA/IC version 16: STATA Corp LLC., College Station, TX).

## Results

**Fig 2:**
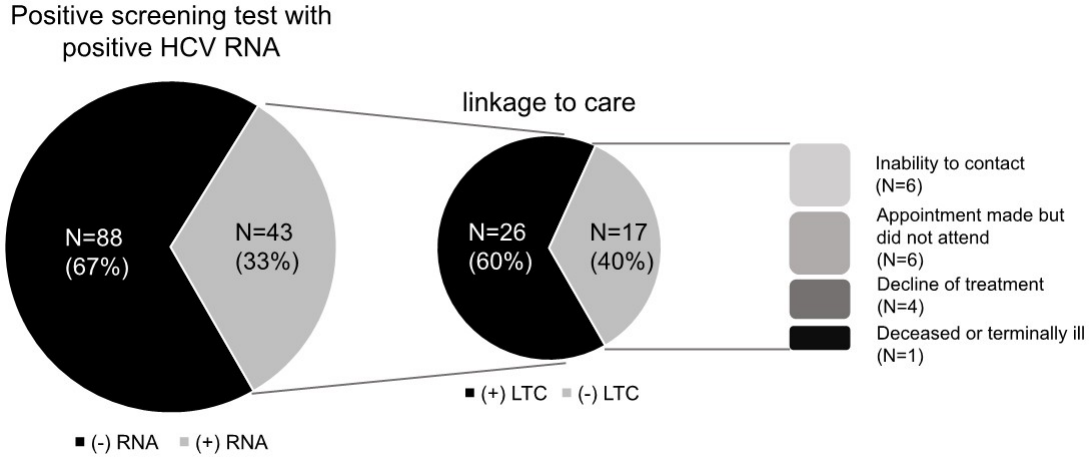
HCV screening and LTC with common barriers.

A total of 4525 patients underwent a screening test, of whom 131 patients (2.90%) were HCV antibody positive, and 43 (0.95%) were HCV RNA positive, indicating that only 33% of patients with a positive screening test had a chronic infection (Fig 2). The rate of chronic infection was higher in males as compared to females (1.34% vs 0.60%, p = 0.01) (Table 1). Of the 43 patients who had a chronic HCV infection, 9 (20.9%) were aware of their infection but were not engaged in treatment. Of the 131 patients who had HCV antibodies, 12 (9.1%) had been treated for chronic infection. Of the 119 patients who had positive HCV antibodies and had not received any treatment for chronic infection, 78 (65.5%) had had a negative HCV RNA test, which can suggest spontaneous clearance. The spontaneous clearance rate was higher in females than males although this did not meet statistical significance (72.3% vs 60.0%, p = 0.17) (Table 1).

**Table 1:** Chronic HCV infection rate, spontaneous clearance rate, and LTC according to patient characteristics.

**Chronic HCV screening**			
Number of tests performed		4525	
Number of patients with positive HCV antibodies		131 (2.90%)	
Number of patients with positive HCV RNA		43 (0.95%)	
**Chronic HCV infection rate**			
Male	Female	p = 0.01	
1.34%	0.60%		
**Spontaneous clearance rate**			
		Univariate analysis	Multivariable logistic regression
Male	Female	p = 0.17	
60.0%	72.3%		
Opioid use	Non-opioid use	p = 0.02	p = 0.03
48.6%	72.0%		
Tobacco use	Non-tobacco use	p = 0.01	p = 0.37
56.6%	80.5%		
**Linkage to care per insurance status**			
Private insurance	Medicare/Medicaid	No insurance	p = 0.06
60.0%	73.3%	0%	

In patients with the positive screening test, 39 (29.8%) had a history of opioid use and 84 (64.1%) had a history of tobacco use. Patients with a history of opioid use had a lower rate of spontaneous clearance than patients without the history (48.6% vs 72.0%, p = 0.02) (Table 1). The prevalence of opioid use in patients with chronic hepatitis C was the highest in the youngest age group (born after 1965: 73.3% vs. born between 1945 and 1965: 33.3% vs. born before 1945: 0%, p = 0.009), reflecting that the main cause of chronic HCV infection in that age population is injection drug use, including opioids. Patients with a history of tobacco use had a lower rate of spontaneous clearance than patients without the history (56.6% vs 80.5%, p = 0.01) (Table 1).

In a multiple logistic regression analysis, history of opioid use and history of tobacco use were included as independent predictors for HCV spontaneous clearance. It did not show significant differences between patients with and without a history of tobacco use (p = 0.37). However, patients with a history of opioid use were significantly less likely to clear HCV spontaneously (p = 0.03) (Table 1). We included sex as the third predictor, but it did not show significant differences between males and females (p = 0.10). History of opioid use remained as a significant factor (p = 0.01) in multiple logistic regression even after adding sex as the third predictor, while history of tobacco use remained nonsignificant (p = 0.56).

Among 43 patients who were diagnosed with chronic hepatitis C, 26 attended the first appointment with medical providers with an LTC rate of 60.5% (Fig 2). Among identifiable reasons for missing the linkage, inability to contact patients after discharge from the ED (not answering phone calls or inability to leave a voice message, n = 6) and not attending the scheduled appointment were (patients did not specify reasons, n = 6) common reasons, followed by decline of treatment (n = 4) and deceased or terminally ill state (n = 1) (Fig 2). Regarding insurance types in newly diagnosed chronic hepatitis C patients, 25 had private insurance, 15 had Medicare/Medicaid, and 3 did not have insurance. The rate of LTC was different according to insurance groups (private insurance: 60.0% vs. Medicare/Medicaid: 73.3% vs. no insurance: 0%, p = 0.06) (Table 1). Although there were similar LTC rates in patients with private insurance and Medicare/Medicaid, none of the patients without insurance were linked to care despite efforts to establish their medical care at regional Federally Qualified Health Centers.

## Discussion

This study emphasizes several clinical and public health implications. First, we identified certain patient characteristics that are related to a lower rate of HCV spontaneous clearance and higher rate of chronic infection. Males had more chronic HCV infections than females, and history of opioid use and history of tobacco use were related to lower HCV spontaneous clearance rate, although history of tobacco use was not a statistically significant factor in multiple logistic regression analysis. This highlights the importance of a higher index of suspicion and targeted screening and treatment in these vulnerable groups. Our results are in line with prior HCV natural history studies, which demonstrated that male sex is related to a lower rate of HCV spontaneous clearance [5,6]. The mechanism of a higher HCV clearance rate in females is uncertain. Regarding tobacco use, it has been reported that the prevalence of smoking in chronic hepatitis C patients is almost three times higher than in patients without chronic hepatitis C [7]. In addition, smoking has been shown to aggravate histologic activity of chronic hepatitis C, independent of alcohol, which can suggest that smoking can worsen hepatocellular injury in individuals who are infected with HCV and impairs spontaneous clearance [8].

Furthermore, a higher rate of past or present opioid use was observed in the younger population with chronic hepatitis C in our data. The CDC reported that acute HCV infection was the highest in the age group 20–29 (2.8 cases per 100,000) in 2017 [9]. During 2006–2012, the combined incidence of acute HCV infection in four states (Kentucky, Tennessee, Virginia and West Virginia) increased 364% among individuals aged 30 years or younger, and the most commonly identified risk factor (73%) was injection drug use [10]. As such, the opioid crisis is likely a major driving factor in persistently high prevalence of chronic hepatitis C in the US. In addition, opioid use likely impairs HCV spontaneous clearance by the patient engaging in unsafe behaviors such as sharing injecting equipment and practicing poor sterilization procedures, as well as high-risk sexual behaviors [11].

Interestingly, HCV spontaneous clearance rate (65.5%) in our study was higher than in previous reported studies [2,5,6]. However, a similar spontaneous clearance rate was observed in a recent study [12]. One of the possible reasons is that it became easier to capture anti-HCV seropositive individuals with concurrent negative HCV RNA test after the CDC updated laboratory criteria for chronic hepatitis C in 2016, which required positive HCV antibodies with reflex to positive HCV RNA [13]. Future studies are expected to show a more accurate spontaneous clearance rate.

In our study, 43 patients were newly identified as having chronic hepatitis C during the study period and 26 patients kept their first appointment with medical providers for further management. Insurance types were closely related to LTC rate and patients without insurance failed to establish care despite social workers’ efforts to make appointments at a regional Federally Qualified Health Centers. We recognize that the most common barrier that can be addressed is the inability to contact patients after discharge from the ED. For future studies, it will be important to obtain and confirm contact information during the first patient encounter.

The ED presents a unique opportunity to screen patients who may not otherwise be engaged in medical care. A recent HCV screening study in four different EDs demonstrated that screening for HCV in the ED can identify a large number of previously unrecognized HCV infections [14]. Interestingly, the prevalence of positive HCV RNA at the combined ED sites was 5.7%, which was significantly higher than the estimated overall US prevalence of positive results for HCV RNA of 0.95%, which was similar to our study (0.95%) [15]. Although screening in the ED has been demonstrated to be a high-yield and effective intervention, a 2014 decision from the US Department of Health and Human Services and Centers for Medicare and Medicaid Services precluding EDs from reimbursement for HCV screening may be a limiting step in HCV screening in the ED [16]. In our study we were able to implement a risk-based screening program for ED patients and established a system to facilitate LTC by using a grant from Gilead Sciences.

Our study has several limitations beside its small sample size and retrospective nature. First, a concurrent positive HCV antibody test and negative HCV RNA test does not necessarily mean spontaneous clearance. It is possible that a positive HCV antibody test is a false positive. The specificity of HCV antibody tests in our institution (VITROS anti-HCV test) is estimated to be 98.22%, which gives us a false positive rate of 1.78%. This cannot be overlooked when prevalence of the disease is low, such as in our case. The clinical significance of false positives has become more meaningful since the CDC broadened the screening criteria [4], which decreased positive predictive value. However, the laboratory department at Englewood Health developed an institutional policy to repeat the HCV antibody test in duplicate if the signal-to-cutoff ratio was between 0.9 and 2.0, which was changed from the previous range of 0.9 to 1.0. If two of the three results are <1.0, the sample is considered negative and if two of the three results are ≥ 1.0, the sample is considered positive, which is followed by RNA test.

Second, we could not identify the timing of HCV infection or clearance in every patient, which would have been ideal in evaluating the natural course of the disease. We accessed the New Jersey Communicable Disease Reporting and Surveillance System and tried to identify the timing of the test results, but the data were often missing. However, we obtained history including self-awareness of the disease and previous treatment history as much as possible to help us understand the disease course in each individual.

## Conclusions

In summary, we demonstrated that a streamlined EMR system for HCV screening and subsequent LTC from the ED can be successfully implemented. Between August 1, 2018 and April 30, 2020, we screened 4,525 patients, identified 43 chronic hepatitis C patients, and linked 26 patients to medical providers. A retrospective chart review showed that history of opioid use and history of tobacco use were related to lower HCV self-clearance and that males are more likely to have chronic HCV infection than females. Furthermore, LTC was closely related to insurance status, and common barriers to LTC were inability to contact the patient after discharge from the ED and patients not attending a scheduled appointment for unclear reasons. These findings emphasize the importance of strategic HCV screening for the vulnerable population and more focused resource distribution for uninsured patients.

## Supporting information

S1 File Patient dataset. (XLSX)

## Abbreviations

CDC: Centers for Disease Control and Prevention
ED: emergency department
EMR: electronic medical record
HCV: hepatitis C virus
LTC: linkage to care
